# Reporting randomised clinical trials of analgesics after traumatic or orthopaedic surgery is inadequate: a systematic review

**DOI:** 10.1186/1472-6904-10-2

**Published:** 2010-01-12

**Authors:** Eva Montané, Antoni Vallano, Xavier Vidal, Cristina Aguilera, Joan-Ramon Laporte

**Affiliations:** 1 Fundació Institut Català de Farmacologia. Clinical Pharmacology Service. Hospital Universitari Vall d'Hebron. Pg Vall d'Hebron, n° 119-129. 08035 Barcelona. Universitat Autònoma de Barcelona. Spain

## Abstract

**Background:**

Several randomised clinical trials (RCTs) of analgesics in postoperative pain after traumatic or orthopaedic surgery (TOS) have been published, but no studies have assessed the quality of these reports. We aimed to examine the quality of reporting RCTs on analgesics for postoperative pain after TOS.

**Methods:**

Reports of RCTs assessing analgesics in postoperative pain after TOS were systematically searched from electronic databases. The quality of reports was assessed using the CONSORT checklist (scoring range from 0 to 22). The quality was considered poor when scoring was 12 or lesser. The publication year and the impact factor of journals were recorded.

**Results:**

A total of 92 reports of RCTs were identified and 69 (75%) scored 12 or lesser in CONSORT checklist (range 5-17). The mean (SD) CONSORT score of all reports was 10.6 (2.7). Missing CONSORT items included primary and secondary outcome measures (11%), the specific objectives and hypothesis definition (12%), the sample size calculation (12%), the dates defining the periods of recruitment (12%), the discussion of external validity of findings (14%), the allocation sequence generation (24%), and the interpretation of potential bias or imprecision of results (25%). There was a little improvement in CONSORT scores over time (r = 0.62; p < 0.001) and with impact factor of journals (r = 0.30; p < 0.001).

**Conclusion:**

Quality of reporting RCTs on analgesics after TOS is poor. Reporting of those RCTs should be improved according to methodological standard checklists in the next years.

## Background

Traumatic and orthopaedic surgery (TOS) is one of the most painful surgical interventions. Evidence on the efficacy of analgesic drugs for pain after TOS is usually obtained from randomised clinical trials (RCTs). However, interpretation of results can be hampered by incomplete data reporting and by low methodological quality of the trial [1]. The quality of the evidence provided by orthopaedic journals about major orthopaedic surgery has been assessed, [2,3] as well as the quality of the RCTs assessing analgesic interventions [4]. We have assessed characteristics of patients included in RCTs on analgesics drugs for pain after TOS, as well as analgesic drugs, outcomes and observation periods in a previous study [5]. Nevertheless, quality in reporting RCTs of analgesics for postoperative pain after TOS has not been systematically assessed. The aim of this study was to examine the quality of reporting RCTs on the efficacy of analgesic drugs in postoperative pain after TOS.

## Methods

### Data sources and searching

A systematic review assessing quality of RCTs of analgesic drugs in pain after TOS was performed. A search from electronic databases PubMed, EMBASE, and The Cochrane Library, and references from identified papers and hand searches were carried out. The search included combinations of the following MeSH terms: "pain, postoperative", "randomised controlled trials", "analgesics", "anti-inflammatory agents, non steroidal", and "orthopaedics".

### Selection criteria

RCTs of analgesic drugs for the control of pain after TOS published from January 1966 to June 2006 were included. The language of the reports was restricted to English, French, Italian, German, and Spanish. Reports of clinical trials in adult patients were included if they were parallel group studies, and if patients had been randomly allocated to the various treatment groups (either opioids, paracetamol, or non-steroidal anti-inflammatory drugs [NSAIDs], combinations of them or placebo), exclusively after TOS, and the route of drug administration was either oral, intramuscular, intravenous, subcutaneous, rectal, or transcutaneous. Only full original articles were considered for inclusion. Case reports, abstracts, and letters were excluded. RCTs on anaesthetics, on preoperative or perioperative drug administration, and on spinal drug administration, and those on patients undergoing disc surgery were all excluded.

### Data abstraction and study characteristics

The following data were extracted from each report: language, year of publication, geographical area where the study was carried out, medical journal, its impact factor, and its medical area. The quality of reporting of each included study in the reports was assessed independently by three evaluators (EM, AV, CA) with CONSORT checklist [6]. The quality in reporting trials was scored according to the revised CONSORT checklist, which includes 22 items assessing the quality of the contents of the report (*Title and Abstract, Introduction, Methods, Results, and Discussion*). Discrepancies between the evaluators were discussed and resolved by consensus.

The quality of RCTs was classified in three categories according to CONSORT score: excellent (≥ 20 items), good (between 13 and 19), and poor (≤ 12).

The impact factor, for each journal where the RCTs were published, was determined from the Science Citations Index (http://www.accesowok.fecyt.es/, last accessed March 23, 2009). The RCTs from journals without a citation index were excluded from the impact factor median and correlation analysis. The RCTs were considered to be from high-impact journals if the citation index was above the median of the journals included in the study, low-impact journals if the citation index was below the median and no-impact journals if they didn't have a citation index. These three categories were used for the comparative analysis between quality scales.

### Statistical analysis

Mean (SD), median (range), and frequencies were used as descriptive statistics according to the variable characteristics. Comparison of the mean overall scores for CONSORT checklist according to compared groups in RCTs (with placebo or without placebo group, and with different compared analgesics) was carried out with ANOVA analysis. The Spearman correlation was used for correlations between the overall scores of each trial in the CONSORT checklist and the year of publication, and the impact factor of the journal where the report had been published. Comparisons between overall score in the CONSORT checklist and impact factor of the journals were carried out with ANOVA analysis; p-values were adjusted using the Bonferroni method. Statistical significance was considered when p-values were less than 0.05. Agreement between evaluators was assessed with intraclass correlation coefficient and 95% confidence intervals (ICC and 95% CI). We accepted the definition of a high level of agreement represented by values of ICC from 0.65 [7]. All analyses were conducted using SPSS 12.0 (SPSS Inc, Chicago IL, USA).

## Results

A total of 326 reports were selected, and 235 were excluded because the studies were not RCTs, orthopaedic surgery was combined with another type of surgery, non analgesic drugs were also assessed, and for other reasons. Ninety-one publications reporting 92 RCTs met inclusion criteria (one publication reported two RCT). Eighty four (91.3%) were published in English, six (6.5%) in French, and two (2.2%) in Italian. The first report had been published in 1971, and 49 (53.3%) had been published after 1990. The articles described 58 RCTs carried out in Europe (63%), 26 in America (26; 28.3%), 5 in Asia (5.4%), and 3 in Africa (3.3%). Reports were published in 46 medical journals from different medical areas, mainly Pharmacology and Therapeutics 32 (35%), and Anaesthesiology 26 (28%). A total of 76 (82.6%) RCTs were published in 34 journals included in the Science Citation Index. The median impact factor was 1.76 (range 0.31 - 7.53). Thirty-six reports had been published in low-impact journals, and 40 in high-impact journals.

The agreement (ICC) between the three evaluators for the overall scores of the CONSORT checklist assessed was 0.77 (95%CI, 0.70 - 0.84). The mean (SD) CONSORT checklist score was 10.5 (2.7). The CONSORT checklist score ranged from 5 to 17. The quality of RCTs was good in 23 (25%) reports and poor in 69 (75%). The details of the scores of the items in the CONSORT checklist are given in Table 1. In the *Methods *section the following items were poorly described: outcomes (ten trials, 10.9%), objectives and hypothesis (11 trials, 12%), sample size calculation (11 trials, 12%), and the sequence generation of randomisation (22 trials, 23.9%). In the *Results *section the dates defining the periods of recruitment and follow-up only were reported in 11 trials (12%). The analysis of outcomes was by intention to treat and the number of participants included in each analysis was reported in 24 trials (26%). The estimated effect size of the outcomes and its precision was described in 40 (43.5%). In the *Discussion *section, the external validity of the trials findings only were commented in 13 trials (14.1%), and the interpretation of the results, taking into account hypothesis, sources of potential bias or imprecision, and the dangers associated with multiplicity of analysis and outcomes was adequately described in 23 trials (25%). Differences in reporting were observed between RCTs that included a placebo control group and those without a placebo group. The mean scores in RCTs with placebo group were higher than in RCTs without placebo group. No differences in reporting were shown related to different analgesics groups compared in RCTs (Table 2).

**Table 1:** Reporting randomised controlled clinical trials according to different items in CONSORT checklist.

Paper section and Topic	Item number	n RCTs (N = 92)	(%)
***Title and abstract***	1	64	(69.6)
***Introduction***			
Background	2	67	(72.8)
***Methods***			
Participants	3	86	(93.5)
Interventions	4	90	(97.8)
Objectives	5	11	(12.0)
Outcomes	6	10	(10.9)
Sample size	7	11	(12.0)
Randomisation			
Sequence generation	8	22	(23.9)
Allocation concealment	9	5	(5.4)
Implementation	10	1	(1.1)
Blinding (masking)	11	75	(81.5)
Statistical methods	12	84	(91.3)
***Results***			
Participant flow	13	62	(67.4)
Recruitment	14	11	(12.0)
Baseline data	15	73	(79.3)
Numbers analysed	16	24	(26.0)
Outcomes and estimation	17	40	(43.5)
Ancillary analyses	18	48	(52.2)
Adverse events	19	88	(95.6)
***Discussion***			
Interpretation	20	23	(25.0)
Generalization	21	13	(14.1)
Overall evidence	22	62	(67.4)

**Table 2:** Quality of reporting according to compared groups in randomised clinical trials.

RCT (n = 92)	CONSORT checklist Mean score (sd)
***Placebo control group***	
	
Yes (n = 42)	11.36 (2.59)
No (n = 50)	9.92 (2.67)
	
*Statistical significance*	p = 0.011
***Analgesics compared***	
	
NSAIDs (n = 46)	10.59 (3.01)
Opioids (n = 10)	11.70 (2.31)
NSAIDs *vs*. Opioids (n = 19)	10.11 (2.28)
NSAIDs + opioids *vs*. NSAIDs and/or opioids (n = 17)	10.41 (2.57)
	
*Statistical significance*	p = 0.511

The correlation between the mean overall CONSORT scores and the year of publication was statistically significant (r = 0.62; p < 0.001), as well as between the mean overall CONSORT scores and the impact factor (r = 0.30; p < 0.001). The mean (SD) CONSORT scores for RCTs published after 2001 (year CONSORT checklist was published) was higher than the mean CONSORT scores for those published previously [14.4 (2) and 10.3 (2.5) respectively; p < 0.0001]. The mean overall score in the CONSORT checklist was higher in high-impact journals. Differences were statistically significant in the mean overall CONSORT score for low and no-impact journals compared to high-impact journals (Table 3).

**Table 3:** Mean (SD) overall scores for CONSORT checklist according to impact factor (IF) of the medical journals.

IF journals	No IF journals (n = 16)	Low IF journals (n = 36)	High IF journals (n = 40)	Total journals (N = 92)
**CONSORT** **Mean (SD)**	8.9 (2.3)†	10.1 (2.1)††	11.6 (3.0)	10.5 (2.7)

† p = 0.007 no IF journals *vs*. high IF journals; †† p = 0.012 low IF journals *vs*. high IF journals

## Discussion

The results of the present study illustrate that the overall quality of reporting of RCTs assessing the efficacy of analgesic drugs after TOS was poor. The main deficiencies were lack of information on methods to define the hypothesis and the outcomes, the sample size calculation, and lack of data on the results of estimated effects size and their precision. These methodological flaws limit their validity and the interpretation of the results, and they may lead to biased findings [1,8]. In fact, few reports adequately interpreted the results of RCTs in discussion section, taking into account sources of potential bias or imprecision, the problems associated with multiplicity of analysis and outcomes or the external validity of findings. Our results are similar to those of other systematic reviews on the methodological quality in orthopaedic-surgery related topics,[3] and on analgesic interventions [4]. However, the results also show that there has been a small and progressive improvement in the quality of reporting of RCTs over time, as described in studies in other therapeutic areas [9-13]. In general, our results indicate that editors and reviewers of journals, as well as readers, should be more aware of the methodological shortcomings in the reports of RCTs evaluating analgesic drugs in postoperative after TOS. We think that most of those methodological deficiencies in reporting RCTs, could be easily corrected and avoided in the future.

Three approaches have been developed to assess the quality of RCTs: one based on specifically relevant individual components (which evaluate selected aspects of trials, such as randomisation or blinding), scales (providing numerical scores of quality), and checklists (that involve lists of items such as CONSORT or Delphi) [14]. The journal impact factor has also been used widely as a quality measure [15]. In our opinion, the CONSORT checklist helps to evaluate quality in a more accurate way because it includes other important methodological items such as the hypothesis description, definition of the primary and secondary outcomes, sample size calculation, and others. On the other hand, we expected that journals with the highest impact factors would have the highest scientific quality [16]. Bath et al. [10] have reported a significant association between the CONSORT score and the impact factor in stroke. When we classified the medical journals according to the impact factor (none, low, or high), the CONSORT score was higher in high-impact journals than non-impact journals. Since the reporting guidelines such as the CONSORT checklist, or the Standards for Reporting in Trials (SORT) -focused on reporting side effects- were published or endorsed as a requirement for publishing, reports of clinical research have improved [17,18]. But in fact, in 2003 only 22% of high impact journals had endorsed it,[19] and there are still many journals in anaesthesiology, pain, or orthopaedic topics that have not done so.

Assessment of the methodological quality and reporting has been carried out in other specialities and/or in other conditions [9-13,20-33]. In general, the quality of reporting of RCTs was low or less than optimal, with more than half of all studies scoring less than half of the scores from the quality scales used with the exception of two studies which were adequately reported [13,32]. However, these studies have used a variety of instruments based on different criteria (ranging from 1 to more than 80 items). The CONSORT checklist and the Jadad scale were the most frequently used, as well as modified versions of both [34]. Therefore, it is not possible to make a direct comparison with the results of our study. Most of the referred studies have assessed clinical trials published in specific medical journals (basically assessing the quality of those journals), while the present study focused on a specific problem.

We limited the bias in the selection and in scoring the methodological quality of RCTs by conducting process in triplicate and independently [35]. As available scales and checklists are heterogeneous in size and complexity, their overall scores can also diverge considerably depending on the evaluator [36,37]. However, our results showed high agreement between evaluators in the overall scores of the quality checklist. The CONSORT checklist is not a quality scale but as it gives recommendations on reporting, it provides an unweighted score of reporting compliance, where some items may be clearly more important than others. Moreover, its relevance can vary depending on the condition being assessed (e.g., blinding is crucial in RCTs on the treatment of pain, but less so on those in the treatment of infectious diseases) [10]. Nevertheless, we have used it because of the lack of a better alternative.

It should also be acknowledged that most of the trials included in this review were published several years ago and may no longer reflect current reporting practices. Furthermore, only published trials were retrieved, and authors or pharmaceutical companies were not contacted. Unpublished trials tend to be of a lower methodological quality, and their results tend to be more biased than those of published trials;[38] therefore, the methodological quality of controlled research on analgesics in TOS may be even worse than described in the present report.

## Conclusions

The quality of reporting RCTs on analgesic drugs after TOS is inadequate, although a modest improvement has been seen over time. Therefore, editors and reviewers of journals, as well as readers, should be more aware of the shortcomings in reporting RCTs on analgesic drugs in postoperative pain after TOS. The quality of reporting RCTs on analgesia in TOS should be improved according to standard methodological checklists in the future.

## Competing interests

The authors declare that they have no competing interests.

## Authors' contributions

EM and AV contributed to conception and design, acquisition of data, analysis and interpretation of data, and have been involved in drafting the manuscript. XV contributed to the analysis and interpretation of data, and has been involved in revising critically the manuscript. CA contributed to acquisition of data and has been involved in revising critically the manuscript. JRL contributed to interpretation of data, and has been involved in drafting the manuscript. All authors read and approved the final manuscript.

## Pre-publication history

The pre-publication history for this paper can be accessed here:

http://www.biomedcentral.com/1472-6904/10/2/prepub
